# Fidaxomicin for *Clostridioides difficile* infection in patients with inflammatory bowel disease: a multicenter retrospective cohort study

**DOI:** 10.1093/ecco-jcc/jjaf056

**Published:** 2025-04-01

**Authors:** Daniele Noviello, María Chaparro, Chiara Viganò, Andreas Blesl, Brigida Barberio, Henit Yanai, Ambrogio Orlando, Rocío Ferreiro-Iglesias, Cristina Bezzio, Alessandra Zilli, Tamás Molnár, Cristian Gheorghe, Francesco Conforti, Tommaso Innocenti, Simone Saibeni, Peter Bossuyt, Raquel Oliveira, Anna Maria Carvalhas Gabrielli, Alessandra Losco, Sophie Vieujean, Enrico Tettoni, Lorena Pirola, Silvia Calderone, Maya Kornowski Cohen, Gabriele Dragoni, Timo Rath, Manuel Barreiro-de Acosta, Edoardo Vincenzo Savarino, Javier P Gisbert, Maurizio Vecchi, Raja Atreya, Flavio Caprioli

**Affiliations:** Department of Pathophysiology and Transplantation, University of Milan, Milano, Italy; Gastrointestinal Unit of Hospital Universitario de La Princesa, Instituto de Investigación Sanitaria Princesa (IIS-Princesa), Universidad Autónoma de Madrid (UAM), Centro de Investigación Biomédica en Red de Enfermedades Hepáticas y Digestivas (CIBEREHD), Madrid, Spain; Division of Gastroenterology, Center for Autoimmune Liver Diseases, Department of Medicine and Surgery, University of Milano-Bicocca, Monza, Italy; European Reference Network on Hepatological Diseases ERN RARE-LIVER, Fondazione IRCCS San Gerardo dei Tintori, Monza, Italy; Division of Gastroenterology and Hepatology, Department of Internal Medicine, Medical University of Graz, 8036 Graz, Austria; Division of Gastroenterology, Department of Surgery Oncology and Gastroenterology DiSCOG, University of Padova, Padova, Italy; Division of Gastroenterology, Rabin Medical Center, Petah Tikva, Israel; Faculty of Medicine, Tel Aviv University, Tel Aviv, Israel; Inflammatory bowel disease Unit, “Villa Sofia-Cervello” Hospital, Palermo, Italy; Gastroenterology Department, Complejo Hospitalario Universitario de Santiago, Santiago de Compostela, A Coruña, Spain; Department of Biomedical Sciences, Humanitas University, Pieve Emanuele (MI), Italy; IBD Center, Department of Gastroenterology, IRCCS Humanitas Research Hospital, Rozzano (MI), Italy; Department of Gastroenterology & Endoscopy, IRCCS San Raffaele Hospital, Milan, Italy; Department of Internal Medicine, University of Szeged, Szeged, Hungary; Center of Gastroenterology and Hepatology, Fundeni Clinical Institute, 022328 Bucharest, Romania; Faculty of Medicine, Carol Davila University of Medicine and Pharmacy, 050474 Bucharest, Romania; Gastroenterology and Endoscopy Unit, Fondazione IRCCS Ca’ Granda Ospedale Maggiore Policlinico, Milan, Italy; Gastroenterology Research Unit, Department of Experimental and Clinical Biochemical Sciences “Mario Serio,” University of Florence, Florence, Italy; IBD Referral Center, Clinical Gastroenterology Unit, Careggi University Hospital, Florence, Italy; IBD Centre, Gastroenterology Unit, Rho Hospital, ASST Rhodense, 20017 Rho, Italy; Imelda GI Clinical Research Center, Imelda General Hospital, Bonheiden, Belgium; Gastroenterology Department, Unidade Local de Saúde do Algarve, Portimão, Portugal; School of Medicine, Life and Health Sciences Research Institute (ICVS), University of Minho, Braga, Portugal; Gastroenterology Unit, ASST Fatebenefratelli-Sacco, L. Sacco Hospital, Milano, Italy; Gastroenterology and Digestive Endoscopy Unit, ASST Santi Paolo e Carlo, Ospedale San Carlo, Milan, Italy; Hepato-Gastroenterology and Digestive Oncology, University Hospital CHU of Liège, Liège, Belgium; Gastroenterology and Digestive Endoscopy Unit, Fondazione Poliambulanza Istituto Ospedaliero, Brescia, Italy; Università Cattolica del Sacro Cuore, Fondazione Policlinico Universitario A. Gemelli IRCCS, Rome, Italy; Division of Gastroenterology, Center for Autoimmune Liver Diseases, Department of Medicine and Surgery, University of Milano-Bicocca, Monza, Italy; European Reference Network on Hepatological Diseases ERN RARE-LIVER, Fondazione IRCCS San Gerardo dei Tintori, Monza, Italy; Inflammatory bowel disease Unit, “Villa Sofia-Cervello” Hospital, Palermo, Italy; Division of Gastroenterology, Rabin Medical Center, Petah Tikva, Israel; Faculty of Medicine, Tel Aviv University, Tel Aviv, Israel; Gastroenterology Research Unit, Department of Experimental and Clinical Biochemical Sciences “Mario Serio,” University of Florence, Florence, Italy; IBD Referral Center, Clinical Gastroenterology Unit, Careggi University Hospital, Florence, Italy; Department of Medicine 1, Universitätsklinikum Erlangen and Friedrich-Alexander-Universität Erlangen-Nürnberg, Erlangen, Germany; Gastroenterology Department, Complejo Hospitalario Universitario de Santiago, Santiago de Compostela, A Coruña, Spain; Division of Gastroenterology, Department of Surgery Oncology and Gastroenterology DiSCOG, University of Padova, Padova, Italy; Gastrointestinal Unit of Hospital Universitario de La Princesa, Instituto de Investigación Sanitaria Princesa (IIS-Princesa), Universidad Autónoma de Madrid (UAM), Centro de Investigación Biomédica en Red de Enfermedades Hepáticas y Digestivas (CIBEREHD), Madrid, Spain; Department of Pathophysiology and Transplantation, University of Milan, Milano, Italy; Gastroenterology and Endoscopy Unit, Fondazione IRCCS Ca’ Granda Ospedale Maggiore Policlinico, Milan, Italy; Department of Medicine 1, Universitätsklinikum Erlangen and Friedrich-Alexander-Universität Erlangen-Nürnberg, Erlangen, Germany; Department of Pathophysiology and Transplantation, University of Milan, Milano, Italy; Gastroenterology and Endoscopy Unit, Fondazione IRCCS Ca’ Granda Ospedale Maggiore Policlinico, Milan, Italy

**Keywords:** fidaxomicin, *Clostridioides difficile*, CDI, inflammatory bowel disease

## Abstract

**Background and aims:**

Inflammatory bowel disease (IBD) patients with *Clostridioides difficile* infection (CDI) are at increased risk of adverse outcomes. Data on fidaxomicin use in IBD remain scarce. We assessed the effectiveness and safety of fidaxomicin for CDI and its impact on IBD outcomes in a large international cohort.

**Methods:**

Adult patients with ulcerative colitis (UC) or Crohn’s disease (CD) treated with fidaxomicin for documented CDI were retrospectively included. The primary outcome was CDI recurrence rate within 8 weeks (*C. difficile* toxin detection and CDI-targeted therapy). Secondary outcomes included sustained response (no CDI-targeted therapy within 12 weeks), IBD therapy escalation, colectomy rate, and all-cause mortality within 30, 90, and 180 days.

**Results:**

Ninety-six patients (57 UC and 39 CD) from 20 IBD centers were included. Most were on advanced IBD therapy. Half had a previous CDI episode, 15% a severe episode. CDI recurrence rate was 10% at week 8, and sustained response 82% at week 12. Compared with patients with previous CDI episode, patients at first episode tended to have a lower recurrence (4.3% vs 16%; *P* = .06) and higher sustained response (91% vs 75%; *P* = .04) rate. IBD therapy escalation was required in 48% with a numerically lower need for patients achieving vs not-achieving sustained response within 30 days (12% vs 20%; *P* = .42). Five UC patients underwent colectomy. One death unrelated to CDI or IBD occurred. One moderate and 5 mild adverse events were reported.

**Conclusions:**

Fidaxomicin was effective and safe in IBD patients with CDI, with greater effectiveness in CDI-naïve patients, potentially influencing short-term IBD outcomes.

## Graphical Abstract

**Graphical Abstract:**
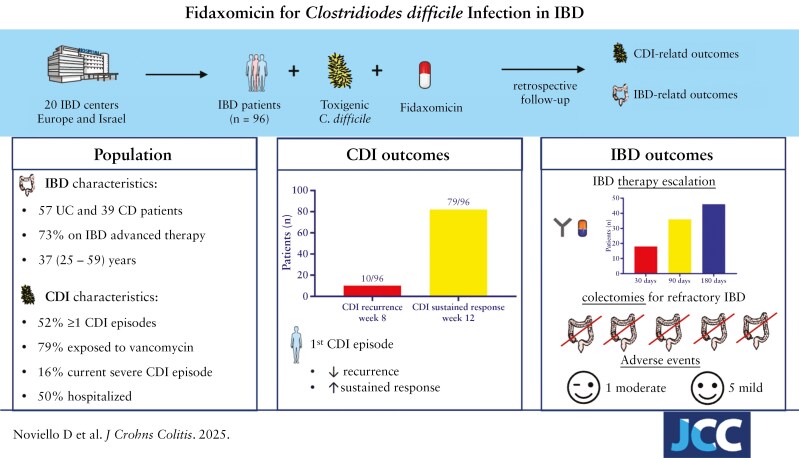

## 1. Background

Patients with inflammatory bowel disease (IBD) have a nearly 5-fold increase in the risk of *Clostridioides difficile* infection (CDI) compared with the general population, harboring an estimated prevalence of 37.3 per 1000 and 10.9 per 1000 among patients with ulcerative colitis (UC) and Crohn’s disease (CD), respectively.^1,2^ Patients with IBD who develop CDI may exhibit atypical clinical features with respect to the general population, including younger age at onset, lack of recent antibiotic use, and community acquisition. Colonic involvement in IBD, biological therapy, and antibiotic use are independent risk factors for CDI among patients with IBD.^3^ In addition, IBD patients are more likely to experience IBD exacerbations requiring therapy escalation, emergency department visits, prolonged hospital stays, and an increased risk of colectomy and mortality.^4–7^ All symptomatic patients with IBD and confirmed CDI by stool testing should receive appropriate antimicrobial therapy. The American College of Gastroenterology suggests vancomycin for a minimum of 14 days in patients with IBD and CDI.^8^ The European Crohn’s and Colitis Organization (ECCO) Guidelines on the Prevention, Diagnosis, and Management of Infections in IBD state that oral vancomycin and fidaxomicin for 10 days are equally effective in treating non-severe CDI.^9^ However, these guidelines are guided by data from the general population and it has not been determined if this also applies to patients with IBD. Some studies specifically investigated the effectiveness of vancomycin in patients with IBD.^10,11^ However, data on fidaxomicin in IBD are very limited, mostly from small, single-center, non-IBD-focused studies.^12–15^ In addition, it remains unexplored whether the resolution of CDI may affect IBD outcomes. We aimed to assess the effectiveness of fidaxomicin for CDI in IBD patients in relation to CDI recurrence rate, the safety profile, and its impact on IBD outcomes in a large retrospective multicenter international cohort study.

## 2. Methods

### 2.1. Study design and patients

This is a retrospective multicenter cohort study involving 20 IBD referral centers across Europe and Israel. We performed a cross-referenced search of fidaxomicin prescriptions with a manual chart review of IBD patients seen at each participating center. Adult patients with an established diagnosis of UC or CD according to the current ECCO guidelines^16^ and a CDI episode, defined as a positive glutamate dehydrogenase antigen test or nucleic acid amplification test for toxigenic *C. difficile* or a toxin enzyme immunoassay, treated with fidaxomicin for at least 7 consecutive days were included (Supplementary Table S1). Patients with unclassified colitis, acute severe UC as per Truelove and Witts criteria,^17^ fidaxomicin therapy for less than 7 consecutive days, toxic megacolon, total abdominal colectomy, and ileal pouch anal anastomosis were excluded.

The date of CDI diagnosis was considered as the baseline. Demographic data, such as gender, age, smoking status, history of malignancy, and comorbidities; and IBD-related characteristics, including phenotype by Montreal Classification, age at diagnosis, disease duration, previous and current IBD treatments, C-reactive protein (CRP), faecal calprotectin, colonoscopy, or sigmoidoscopy up to 8 weeks prior to CDI were collected. Furthermore, CDI-related characteristics, such as recent hospitalization, systemic antibiotic use up to 8 weeks prior to CDI, proton pump inhibitors intake, number of CDI episodes, disease severity according to Zar criteria,^18^ colonoscopy or sigmoidoscopy findings or computed tomography features of the colon within one week of CDI diagnosis, complete blood count, serum creatinine, and albumin at baseline; intensity of care at the time of CDI diagnosis and during clinical management, such as outpatient, inpatient, or intensive care unit admission were extracted from patients’ clinical records. Study data were pseudo-anonymized and managed using an electronic data capture tool hosted at Fondazione IRCCS Ca’ Granda Ospedale Maggiore Policlinico (REDCap, Research Electronic Data Capture).^19^

### 2.2. Outcomes

The primary outcome was CDI recurrence rate, defined as *C. difficile* toxin detection and treatment with any antimicrobial directed at CDI or faecal microbial transplant within 8 weeks. Secondary outcomes included sustained response rate defined as no CDI treatment for 12 weeks, IBD therapy escalation rate defined as induction with an IBD advanced therapy, colectomy rate, and all-cause mortality within 30, 90, and 180 days. IBD- and CDI-related characteristics were analyzed through univariate analysis to investigate potential associations with CDI recurrence. The safety profile of fidaxomicin in IBD patients was assessed through a prespecified list of adverse events including dizziness, rash, chills, headache, nausea, vomiting, increased alanine aminotransferase, increased aspartate aminotransferase, hyperuricemia, leukopenia, hypercalcemia, hypocalcaemia, hyperglycaemia, hyponatremia, hypophosphatemia, neutropenia, lymphopenia, thrombocytopenia, and increased International Normalized Ration (INR), and any other adverse events were collected. The severity of adverse events (mild, moderate, or severe) was assigned according to the evaluation of the local investigator.

### 2.3. Statistics

Standard descriptive statistics were used to analyze patients’ characteristics. Continuous variables were described as median and interquartile range (IQR). Categorical variables were described as the number of cases and proportions. Comparisons between variables were performed by Chi-squared, Fisher exact test, and Mann–Whitney U tests. All statistical analysis was performed by Stata software (Stata Statistical Software: Release 18, StataCorp LLC).

### 2.4. Ethical considerations

The study was performed in accordance with the Declaration of Helsinki, Good Clinical Practice, and applicable regulatory requirements. The study was approved by the Ethics Committee of the coordinating center (Comitato Etico Milano Area 2: 3565) and, thereafter, by all the participating centers.

## 3. Results

### 3.1. Baseline characteristics of the patients

All clinical records of IBD patients in a regular follow-up at each participating IBD center up to September 2024 were retrospectively reviewed. Overall, 99 IBD patients with CDI treated with fidaxomicin were identified between 2013 and 2024 (90 cases from 2019 onward). Three patients were excluded due to incomplete data. Ninety-six patients, 57 with UC and 39 with CD, met the inclusion criteria and were included. IBD characteristics are summarized in Table 1. Most patients had UC (*n* = 57, 59%), median disease duration was 4 (IQR: 1*-*10) years, and 70 (73%) were receiving an advanced therapy. Among patients with an objective IBD disease activity up to 8 weeks prior to CDI diagnosis, 46/70 (66%) had CRP ≥ 1 mg/dL, 37/48 (77%) had a faecal calprotectin ≥ 250 μg/g, 16/20 (80%) a Mayo endoscopic subscore ≥ 2, and 5/8 (63%) a Simple endoscopic score for CD ≥ 7. Table 2 summarizes baseline and CDI characteristics. The median age was 37 (IQR: 25*-*59), and 47 (48%) patients were female. The analyzed episode was the first episode of CDI in 46 (48%) patients, the first recurrence in 29 (31%) patients, and a second and more recurrences in 20 (21%) patients. The timeframe between the previous and the current CDI episode was 2.7 (IQR: 1.6*-*10.3) months. Specifically, 17 (18%) patients had the previous CDI in the previous 2 months. Regarding prior treatments for CDI, most patients had received vancomycin 44 (59%) at standard—125 mg 4 times daily for 10-14 days—or 15 (20%) at extended-pulsed—if treatment lasted over 14 days—dose regimen (Supplementary Figure S1). About a third of patients had at least one additional CDI risk factor, such as recent hospitalization, proton pump inhibitors, and antibiotics use. Most patients (55%) were diagnosed in an outpatient setting, while the remaining were diagnosed during hospitalization, either due to IBD-related issues (38%) or unrelated conditions (6%). Half of the patients were hospitalized, and 16% fulfilled the definition of a severe CDI episode. In the 20 patients with an abdominal computed tomography within 1 week of CDI diagnosis, increased bowel wall thickness and bowel wall enhancement were predominantly reported. Ulcers and spontaneous bleeding were the most common endoscopic findings within 1 week of CDI diagnosis, while pseudomembranous were observed in only 2 patients (13%).

**Table 1.** Characteristics of inflammatory bowel disease patients at CDI diagnosis.

	All patients (*n* = 96)	Ulcerative colitis (*n* = 57)	Crohn’s disease (*n* = 39)	*P*
Age at onset, *n* (%)				.09
- < 16 years	14 (14.6%)	12 (21.1%)	2 (5.1%)	
-17 40 years	56 (58.3%)	30 (52.6%)	26 (66.7%)	
- > 40 years	26 (27.1%)	15 (26.3%)	11 (28.2%)	
Disease extension, *n* (%)				-
-Proctitis	-	6 (10.6%)	-	
-Left-side	-	11 (19.3%)	-	
-Extensive	-	40 (70.2%)	-	
Disease location, *n* (%)				-
-Ileal	-	-	3 (7.7%)	
-Colonic	-	-	11 (28.2%)	
-Ileocolonic	-	-	25 (64.1%)	
Disease behavior, *n* (%)				-
-Inflammatory	-	-	26(66.7%)	
-Stricturing	-	-	8(20.5%)	
-Penetrating	-	-	3(7.7%)	
Disease duration, m	45.1 (11.3*-*115.9)	33.8 (17.5*-*107.4)	55.6 (5.1*-*136.9)	1
Prior Crohn’s disease-related surgery, *n* (%)	-	-	11	-
Body mass index	21.7 (19.7*-*23.9)	21.7 (19.7*-*23.9)	21.6 (19.7*-*23.5)	.91
Family history of IBD, *n* (%)	9 (9.2%)	5 (8.6%)	4 (10.0%)	.91
Appendectomy, *n* (%)	11 (12.2%)	3 (5.2%)	8 (20.5%)	.07
Smoking, *n* (%)				<.01
-never	56 (58.3%)	41 (71.9%)	15 (38.5%)	
-former	18 (18.8%)	5 (8.7%)	13 (33.3%)	
-active	17 (17.7%)	7 (12.3%)	10 (27.6%)	
Prior systemic steroids cycles	2.2 ± 2.3	2.2 ± 1.9	2.2 ± 2.9	.82
Failed azathioprine, *n* (%)	39 (40.2%)	27 (47.4%)	12 (30.0%)	.10
Failed methotrexate, *n* (%)	4 (4.1%)	2 (3.5%)	2 (5%)	.48
Naïve to advanced therapies, *n* (%)	26 (27.1%)	16 (28.1%)	10 (25.6%)	.79
Previous advanced therapy use, *n* (%)				
-Infliximab	46 (65.7%)	28 (68.3%)	18 (62.1%)	
-Adalimumab	26 (37.1%)	9 (21.9%)	18 (62.1%)	
-Golimumab	2 (2.9%)	2 (4.9%)	-	
-Vedolizumab	35 (50.0%)	24 (58.5%)	11 (37.9%)	
-Ustekinumab	22 (31.4%)	12 (29.2%)	10 (34.5%)	
-Risankizumab	4 (%)	-	5 (17.2%)	
-Tofacitinib	5 (%)	4 (9.8%)	-	
-Filgotinib	1 (%)	1 (2.4%)	-	
-Upadacitinib	3 (%)	3 (7.3%)	1 (3.4%)	
-Etrasimod	2 (%)	1 (2.4%)	-	
Number of exposure to advanced therapy lines				.12
-First	25 (35.7%)	15 (36.6%)	10 (33.3%)	
-Second	25 (35.7%)	15 (36.6%)	11 (36.7%)	
-Third	9 (12.9%)	5 (12.2%)	4 (13.3%)	
-Fourth	11 (15.7%)	6 (14.6%)	5 (16.7%)	
C-reactive protein*, mg/dL	2.3 (0.5*-*7.7)	2.1 (0.5*-*7.7)	2.7 (0.6*-*6.0)	.74
Calprotectin*, μg/g	660 (262*-*1400)	732 (490*-*1080)	624 (123*-*1640)	.58
Mayo Endoscopic Subscore^*^				-
-1	-	4 (20.0%)	-	
-2	-	8 (40.0%)	-	
-3	-	8 (40.0%)	-	
Simple Endoscopic Score for Crohn’s Disease^*^				-
-mild	-	-	3 (37.5%)	
-moderate	-	-	2 (25.0%)	
-severe	-	-	3 (37.5%)	
Concomitant** steroids, *n* (%)				.24
-Methylprednisolone	11 (33.3%)	9 (40.9%)	2 (18.2%)	
-Prednisone	17 (51-5%)	10 (45.5%)	7 (63.6%)	
-Budesonide	3 (9.1%)	1 (4.5%)	2(18.2%)	
-Beclomethasone dipropionate	2 (6.1%)	2 (9.1%)	-	
Concomitant** immunomodulator, *n* (%)	5 (5.2%)	4 (7.0%)	1 (2.5%)	.64
Concomitant** advanced therapy, *n* (%)				.11
-Infliximab	13 (23.2%)	8 (22.2%)	5 (25.0%)	
-Adalimumab	6 (10.7%)	2 (5.6%)	4 (20.0%)	
-Golimumab	1 (1.8%)	1 (2.8%)	-	
-Vedolizumab	17 (30.4%)	12 (33.3%)	5 (25.0%)	
-Ustekinumab	9 (16.1%)	6 (16.7%)	3 (15.0%)	
-Risankizumab	2 (3.6%)	-	2	
-Tofacitinib	3(5.4%)	3 (8.3%)	-	
-Filgotinib	1 (1.8%)	1 (2.8%)	-	
-Upadacitinib	3 (5.4%)	2(5.6%)	1 (5.0%)	
-Investigational drug	1 (1.8%)	1 (2.8%)	-	

^*^Up to 8 weeks prior to CDI diagnosis; **at CDI diagnosis.

**Table 2.** Baseline characteristics and characteristics of *Clostridioides difficile* infection.

	All patients (*n* = 96)	Ulcerative colitis (*n* = 57)	Crohn’s disease (*n* = 39)	*P*
Age at CDI diagnosis, years	37 (25*-*59)	35 (24*-*58)	40 (26*-*62.5)	.45
Female, *n* (%)	47 (48.0%)	27 (46.6%)	20 (50.0%)	.74
Number of previous CDI episodes, *n* (%)				.80
-None	46 (48.4%)	25 (44.6%)	21 (53.9%)	
-One	29 (30.5%)	18 (32.1%)	11 (28.2%)	
-Two	14 (14.7%)	9 (16.1%)	5 (12.8%)	
-Three or more	6 (6.3%)	4 (7.1%)	2 (5.1%)	
Timeframe between last and current CDI episode, m	2.7 (1.6*-*10.3)	2.5 (1.6*-*12.5)	3.3 (1.6*-*6.9)	.76
Therapy for previous CDI episodes, *n* (%)				
-Metronidazole	12 (16%)	6 (12.2%)	6 (23.1%)	
-Vancomycin standard dose	44 (58.7%)	29 (59.2%)	15 (57.7%)	
-Vancomycin extended-pulsed	15 (20%)	13 (26.5%)	2 (7.7%)	
-Fidaxomicin	3 (4%)	0	3 (11.5%)	
-Fecal microbial transplantation	1 (1.3%)	1 (2%)	0	
Hospitalization up to 8 weeks prior to current CDI episode, *n* (%)	37 (38.5%)	17 (30.4%)	20 (51.3%)	.04
-IBD-related	25 (67.6%)	13 (76.5%)	12 (60%)	
-CDI-related	8 (21.6%)	3 (17.7%)	5 (25%)	
-Infectious disease-related	1 (2.7%)	0	1 (5%)	
Proton pump inhibitors, *n* (%)	26 (27.4%)	13 (23.6%)	12 (30.8%)	.34
Antibiotics use in the 3 months prior to current CDI episode, *n* (%)	33 (35.9%)	15 (28.3%)	18 (47.4%)	.16
-Clindamycin	2	1	1	
-Cephalosporins	9	4	5	
-Penicillin	9	2	7	
-Fluoroquinolones	9	4	5	
-Tetracycline	1	1	0	
Current severe CDI episode, *n* (%)	15 (15.5%)	8 (14.0%)	7 (17.9%)	.60
Care intensity at diagnosis, *n* (%)				.84
-outpatients	53 (55.2%)	31 (54.4%)	22 (56.4%)	
-inpatients for IBD-related condition	37 (38.6%)	23 (40.4%)	14 (35.9%)	
-inpatients for non IBD-related condition	6 (6.2%)	3 (5.2%)	3 (7.7%)	
Care intensity during management, *n* (%)				.68
-outpatients	48 (49.5%)	27 (47.4%)	21 (52.5%)	
-inpatients	48 (49.5%)	29 (50.9%)	19 (47.5%)	
Leukocytes, WBC/mm^3^	9050 (6375*-*13,715)	9050 (6430*-*12,435)	9100 (6030*-*14,260)	
Albumin,	3.8 (3.1*-*4.3)	3.8 (3.24*-*4.3)	3.9 (3.1*-*4.2)	
Creatinine	0.8 (0.68*-*1.0)	0.88 (0.7*-*1.0)	0.74 (0.66*-*0.9)	
Abdominal computed tomography within 1 week of CDI diagnosis, *n* (%)	20	10	10	
-Increased bowel wall thickness	14 (70.0%)	8 (80.0%)	6 (60.0%)	.33
-Bowel wall enhancement	8 (40.0%)	6 (60.0%)	2 (20.0%)	.07
-Bowel ditation	3 (15.0%)	1 (10.0%)	2 (20.0%)	.53
Endoscopic findings within 1 week of CDI diagnosis, *n* (%)	15	8	7	
-Pseudomembranes	2 (13.3%)	1 (12.5%)	1 (14.3%)	.92
-Ulcers	13 (86.7%)	7 (87.5%)	6 (87.7%)	.92
-Spontaneous bleeding	6 (40.0%)	4 (50.0%)	2 (28.6%)	.40

### 3.2. Effectiveness of fidaxomicin for CDI in relation to CDI recurrence rates

**Figure 1.**
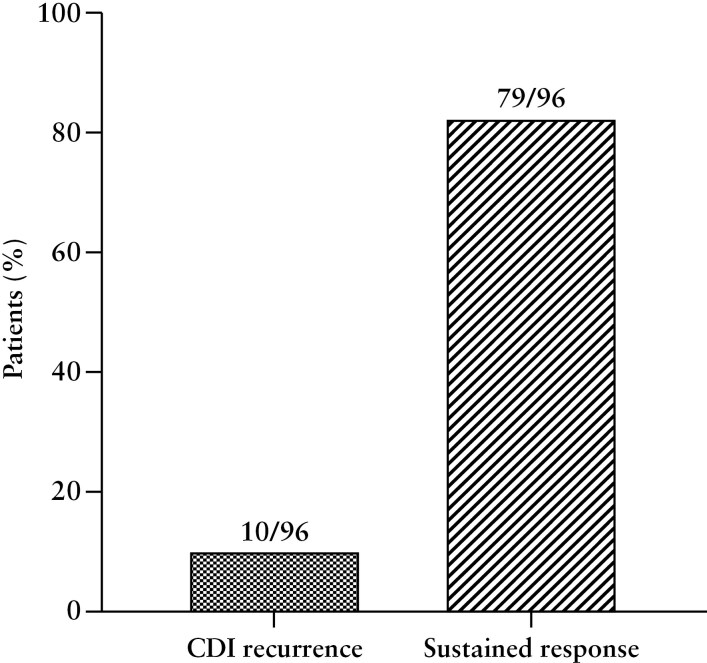
CDI recurrence rate at week 8 and sustained response rate at week 12.

Most patients 83/96 (86%) received a fidaxomicin standard dose regimen (200 mg twice daily for 10 days), 9 received extended-pulsed dosing (200 mg twice daily for days 1-5, then 200 mg on alternate days for days 7-25), 2 received fidaxomicin 200 mg twice daily for 7 days, 2 for 14 days, and 1 for 15 days. CDI recurrence occurred in 10 (10%) patients at week 8, while 79 (82%) patients achieved a sustained response until week 12 (Figure 1). Female sex, but no other demographic characteristic, was associated with a reduced risk for CDI recurrence (Table 3). No IBD-related characteristic, including IBD type and phenotype, exposure to and type of IBD advanced therapy, steroid use at CDI diagnosis, was associated with CDI recurrence (Table 3). Patients at first CDI episode tended to have a lower recurrence rate (4% vs 16%; *P* = .06) and had a significantly higher sustained response (91% vs 75%; *P* = .04) rate compared with patients with previous CDI episode. No difference was found between patients with mild-moderate and severe CDI episodes in terms of CDI recurrence (10% vs 13%; *P* = .85) and sustained response (83% vs 87%; *P* = .87). The timeframe between the previous and current CDI episode, the type of previous therapy for CDI, care intensity, the severity of the current episode, increased bowel wall thickness, and ulcers at endoscopy were not associated with CDI recurrence (Table 3).

**Table 3.** Characteristics of patients with and without CDI recurrence.

	CDI recurrence (*n* = 17)	CDI recurrence- free (*n* = 79)	*P-*value
Age at CDI diagnosis, years	43 (23*-*58)	35 (25*-*62)	.57
Female	3 (17.7%)	43 (54.4%)	.006
BMI	20.9 (20.4*-*24.3)	21.7 (19.6*-*23.4)	.53
IBD disease duration, m	28 (10.8*-*74.4)	46.7 (11.3*-*136.9)	.38
Age at IBD onset			.02
- < 16 years	6 (35.3%)	8 (10.1%)	
-17*-*40 years	6 (35.3%)	50 (63.3%)	
- > 40 years	5 (29.4%)	21 (26.6%)	
UC disease extension			.17
-Proctitis	0	6 (13.3%)	
- Left-side	1 (8.3%)	10 (22.2%)	
-Extensive	11 (91.7%)	29 (64.4%)	
CD disease location			.79
-Ileal	0	3 (3.8%)	
-Colonic	2 (11.7%)	9 (11.4%)	
-Ileocolonic	3 (17.7%)	22 (27.9%)	
-Isolated upper disease	0	1 (1.3%)	
CD disease behavior			.99
-Inflammatory	4 (23.5%)	22 (27.9%)	
-Stricturing	1 (5.9%)	7 (8.9%)	
-Penetrating	0	3 (3.8%)	
Crohn’s disease-related surgery	1	9	
Family history of IBD	1 (5.9%)	8 (10.1%)	.57
Appendectomy	1 (5.9%)	10 (12.7%)	.65
Smoking			.54
-Never	12 (70.6%)	44 (55.7%)	
-Former	2 (11.8%)	16 (20.3%)	
-Active	3 (17.7%)	14 (17.7%)	
Concomitant steroid use	12 (70.6%)	51 (64.6%)	.64
Systemic steroids cycles	32.5 (25*-*60)	25 (15*-*40)	
Failed azathioprine	4 (23.5%)	35 (44.3%)	.11
Failed methotrexate	0	4 (5.1%)	.34
Naïve to advanced therapies	3 (17.6%)	23 (29.1%)	.55
Concomitant advanced therapy			.88
-Infliximab	4 (30.8%)	9 (20.9%)	
-Adalimumab	1 (7.7%)	5 (11.6%)	
-Golimumab	0	1 (2.3%)	
-Vedolizumab	2 (15.4%)	15 (34.9%)	
-Ustekinumab	3 (23.1%)	6 (13.9%)	
-Risankizumab	1 (7.7%)	1 (2.3%)	
-Tofacitinib	1 (7.7%)	2 4.6 (%)	
-Filgotinib	0	1 (2.3%)	
-Upadacitinib	1 (7.7%)	2 (4.6%)	
-Investigational drug	0	1 (2.3%)	
Mayo endoscopic score			.24
-Mild	1 (14.3%)	3 (17.7%)	
-Moderate	1 (14.3%)	8 (47.1%)	
-Severe	5 (71.4%)	6 (35.3%)	
Hospitalization up to 8 weeks prior to CDI diagnosis	6 (35.3%)	31 (39.7%)	.7
Timeframe between last and current CDI episode, m	5.8 (1.7*-*13.9)	2.2 (1.5*-*8.1)	.19
Therapy for previous CDI episodes, *n* (%)			.69
-Metronidazole	0	1 (2.8%)	
-Vancomycin standard dose	7 (58.3%)	23 (63.9%)	
-Vancomycin extended-pulsed	5 (41.7%)	9 (25.0%)	
- Fidaxomicin	0	2 (5.6%)	
-Fecal microbial transplantation	0	1 (2.8%)	
Care intensity			.29
-Outpatients	6 (37.5%)	42 (53.2%)	
-Inpatients	10 (62.5%)	37 (46.8%)	
Current severe CDI episode	2 (11.8%)	13 (16.5%)	.63
Increased bowel wall thickness	5 (25%)	9 (45%)	.26
Ulcers at endoscopy	4 (26.7%)	9 (60.0%)	.99

### 3.3. CDI resolution through fidaxomicin influences IBD outcomes

Induction treatment with an IBD advanced therapy was required in almost half of the patients: 18 (19%), 18 (19%), and 10 (10%) patients within 30, 90, and 180 days, respectively. Of note, 10 patients were naïve to any IBD advanced therapy. These patients started predominantly anti-TNF, while anti-TNF- and vedolizumab-experienced patients initiated anti-IL12/IL23 or -IL23p19 antibody therapy (Supplementary Figure S2). Patients achieving, compared with not achieving, CDI sustained response showed a numerically lower need for IBD therapy escalation within 30 days, with 2 (12%) vs 16 (20%) (*P* = .42). No difference was found between patients with mild-moderate and severe CDI episodes (48% vs 47%; *P* = .92). Overall, 5 (5%) UC patients underwent colectomy for IBD-refractory disease activity within 96 (30*-*117) days after CDI diagnosis, despite advanced IBD therapy induction in 2 patients and without any recurrence of CDI. A 79-year-old UC patient with congestive heart failure, peripheral vascular disease, and atopic dermatitis died of urosepsis 107 days after CDI diagnosis, unrelated to the CDI episode or UC disease control.

### 3.4. Safety profile of fidaxomicin in IBD patients

Adverse events related to the treatment with fidaxomicin were documented in 5 patients (Table 4). Mild episodes of cutaneous rash (1), nausea (1), conjunctivitis (1), asthenia (1), hypokalaemia (1), and hypocalcaemia (1) were reported. One episode of moderate nausea was reported. No adverse events led to treatment discontinuation. No previously unreported adverse events were identified.

**Table 4.** Overview of adverse events.

	Fidaxomicin-treated IBD patients with CDI (*n* = 96)
Dizziness	0
Cutaneous rash	1
Chills	0
Headache	0
Nausea	1 (mild), 1 (moderate)
Vomiting	
Conjunctivitis	1
Asthenia	1
Laboratory abnormalities	
-Hyperuricemia	0
-Increased AST and/or ALT	0
-Leukopenia	0
-Azotemia	0
-Hypercalcemia	0
-Hypocalcemia	1
-Hyperglycemia	0
-Hyponatremia	0
-Hypophosphatemia	0
-Hypokalemia	1
-Neutropenia	0
-Lymphopenia	0
-Thrombocytopenia	0
-Increased International Normalized Ration	0

## 4. Discussion

This international multicenter cohort study provided the largest dataset so far collected on the real-life effectiveness and safety of fidaxomicin treatment in IBD patients with CDI. Our results showed that fidaxomicin was effective and safe for CDI resolution, with greater effectiveness for first episodes than recurrent ones which is comparable with non-IBD cohorts.^20^ Furthermore, CDI resolution might influence short-term IBD outcomes. The safety profile was consistent with that of fidaxomicin application in the general population.^21,22^

In the absence of randomized control trials (RCTs) specifically designed to investigate the effectiveness and safety of CDI-targeted therapies in IBD patients with CDI, reliance on real-world evidence from observational studies is extremely valuable in filling knowledge gaps. In this specific scenario, 2 case series investigated the effectiveness of fidaxomicin in subjects at-risk of CDI, including IBD patients, or 2 in IBD patients alone. However, no definitive conclusions can be drawn due to several limitations. A small sample size (up to 29 IBD patients), heterogeneous definitions of recurrence (diarrhoea, diarrhoea requiring hospital admission, diarrhoea and *C. difficile* toxin, diarrhoea, and any antibiotic treatment targeting CDI), and a heterogeneous follow-up timeframe (30 days, 8-12 weeks, 180 days).^12–15^ Our multicenter cohort study was designed to overcome these limitations with a large sample size, a stringent recurrence definition, and a follow-up timeframe consistent with RCTs.

The observed recurrence rate of 10% aligns with the lower end of the 7%*-*30% range reported in previous studies.^12–15^ This relatively low rate could be partly attributable to the strict recurrence definition, including the need for a CDI-targeted therapy, and the proportion of patients at first CDI episode. Notably, this rate is consistent with fidaxomicin data from the general population, predominantly first CDI episode, where recurrence required likewise a CDI-targeted treatment. No demographic characteristic, except for sex, was associated with a CDI recurrence risk. Specifically, female sex was associated with a reduced risk for CDI recurrence. Although epidemiological studies identified the inverse association between female sex and multiply recurrent CDI (defined as at least 3 courses of CDI antibiotics),^23^ no sex-related effect was reported in fidaxomicin RCTs among the general population.^21,22^ Notably, patients who received fidaxomicin for the first CDI episode experienced fewer recurrences than those with prior CDI episodes. This observation is consistent with data from the general population.^21,22^ However, applying fidaxomicin at the initial CDI occurrence may be particularly beneficial to IBD patients.

In fact, most studies associate the occurrence of CDI in IBD patients with an increased need for treatment optimization, colectomy, and mortality.^4–7^ In our cohort, a significant proportion (48%) of patients required IBD therapy escalation within 180 days after CDI diagnosis compared with a literature rate ranging between 21% and 47%.^4,11,24,25^ The high rate in our cohort could be partly attributable to the baseline IBD characteristics as most patients were already on advanced therapy, and some were on a second or subsequent line of therapy, compared with previous studies. Interestingly, patients achieving CDI resolution showed a numerically lower need for a novel advanced therapy within 30 days. Our results suggest that achieving sustained CDI resolution may potentially influence short-term IBD outcomes. This finding underscores the potential indirect benefit of CDI resolution on IBD disease control. Employing fidaxomicin at the initial occurrence of CDI may offer particular benefit to IBD patients, as it could reduce the vicious cycle of repeated CDI relapses and preempt the need for escalating both CDI- and IBD-related therapies. In fact, the management of steroids and advanced therapies for IBD disease control during a CDI episode can be challenging. According to the ECCO Guidelines on the Prevention, Diagnosis, and Management of Infections in IBD, the use of immunosuppressants can be maintained after careful risk-benefit evaluation and clinical judgment.^9^ The American Gastroenterological Association clinical practice guidelines recommend initiation of corticosteroids or immunosuppressive therapy 3*-*4 days after persistent symptoms of colitis despite appropriate antimicrobial therapy for CDI.^26^ Notably, corticosteroid use has been linked to a higher risk of colectomy in some cohorts, while biologic therapy may have a protective effect.^27,28^ However, patients receiving 2 or more immunosuppressive medications may face a higher risk of severe complications such as megacolon, bowel perforation, and mortality.^29^ A personalized approach, with vigilant monitoring and prompt therapy modifications, is essential for optimal care.

The observed colectomy rate (5%) aligns with the lower end of the 2%*-*48% range reported in IBD patients with CDI during hospitalization or up to 1-year follow-up.^4,10,11,15,24,30–32^ In our cohort, no colectomy was directly attributed to CDI, with the earliest surgery occurring 1 month after CDI diagnosis.

Fidaxomicin was well tolerated in our cohort. No AE led to treatment discontinuation, and no previously unrecognized safety signals emerged. This aligns with the established safety profile of fidaxomicin reported in general population studies and small IBD-focused case series.

Logistical and economic challenges regarding fidaxomicin use emerge in our cohort, as a substantial proportion of patients were hospitalized and repeatedly treated with metronidazole or vancomycin even for CDI recurrences. In certain countries, reimbursement for fidaxomicin requires hospital admission, limiting its use primarily to severe cases managed in inpatient settings. These inconsistencies across countries highlight the need for standardized reimbursement policies to ensure equitable access to any effective treatment.

Preventive strategies also play a crucial role in managing patients with multiple CDI relapses. Current guidelines recommend that patients in the general population who experience a second or subsequent recurrence of CDI, or those with a history of CDI and a high risk of recurrence, be treated with FMT or suppressive oral vancomycin therapy.^8^ Additionally, recent subgroup analyses suggest that a microbiota-based live biotherapeutic product is both safe and effective in preventing CDI recurrence following standard-of-care antimicrobials in patients with IBD—though it is not yet approved in Europe.^33^

This study comes with limitations of retrospective observational studies and some that should be acknowledged. The administration of varying fidaxomicin regimens may have influenced treatment outcomes, although almost all regimes adhered to the fidaxomicin data sheet. Notably, recent evidence suggests that also a shorter duration of treatment may still be effective.^34^ A few patients were tested exclusively through antigen or amplification test, potentially including colonization only. However, this is consistent with real-world studies on CDI.^14,15^ The absence of a comparator group restricts effectiveness comparison to other treatments, such as vancomycin. Heterogeneity regarding recurrence definition and follow-up timeframe also concerns vancomycin studies in IBD and limits indirect comparison.^10,11^ In addition, even a direct comparison through propensity score between fidaxomicin and vancomycin effectiveness in retrospective observational studies would be almost unfeasible due to the considerable challenges in accounting for the numerous variables associated with CDI and IBD. Thus, this large multicenter cohort provides a representative glimpse into clinical practice across Europe and Israel with a comprehensive assessment of both CDI and IBD disease characteristics, treatment patterns, and outcomes. Prospective controlled studies should confirm our findings and investigate optimal treatment positioning.

In conclusion, fidaxomicin is effective and safe for the resolution of CDI in IBD patients, with greater effectiveness for first episodes. The CDI resolution might reduce the short-term need for a novel advanced IBD therapy.

## Supplementary Material

jjaf056_suppl_Supplementary_Figure_S1

jjaf056_suppl_Supplementary_Figure_S2

jjaf056_suppl_Supplementary_Table_S1

## Data Availability

The data underlying this article will be shared on reasonable request to the corresponding author.
