# Supplementary figures and images for: Fidaxomicin for Clostridioides difficile infection in patients with inflammatory bowel disease: a multicenter retrospective cohort study
Source: J Crohns Colitis. 2025 Apr 1;19(5):jjaf056. doi: 10.1093/ecco-jcc/jjaf056 (PMC12060865; doi:10.1093/ecco-jcc/jjaf056)

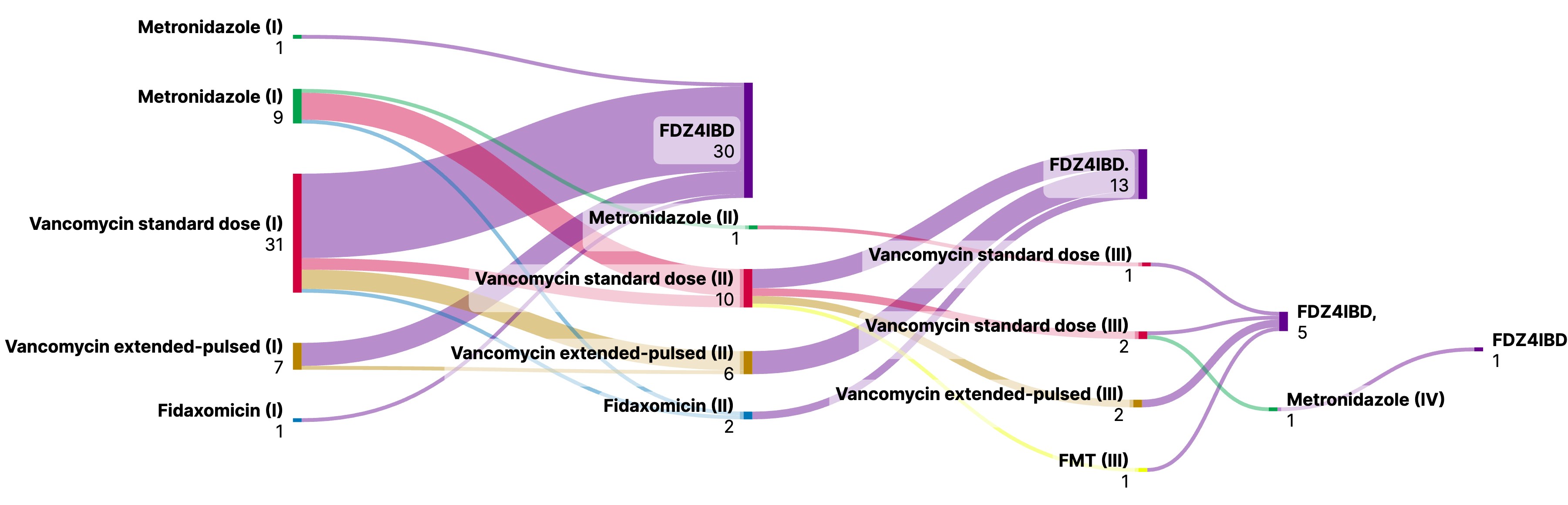

Supplement: jjaf056_suppl_Supplementary_Figure_S1 [file jjaf056_suppl_supplementary_figure_s1.jpeg]

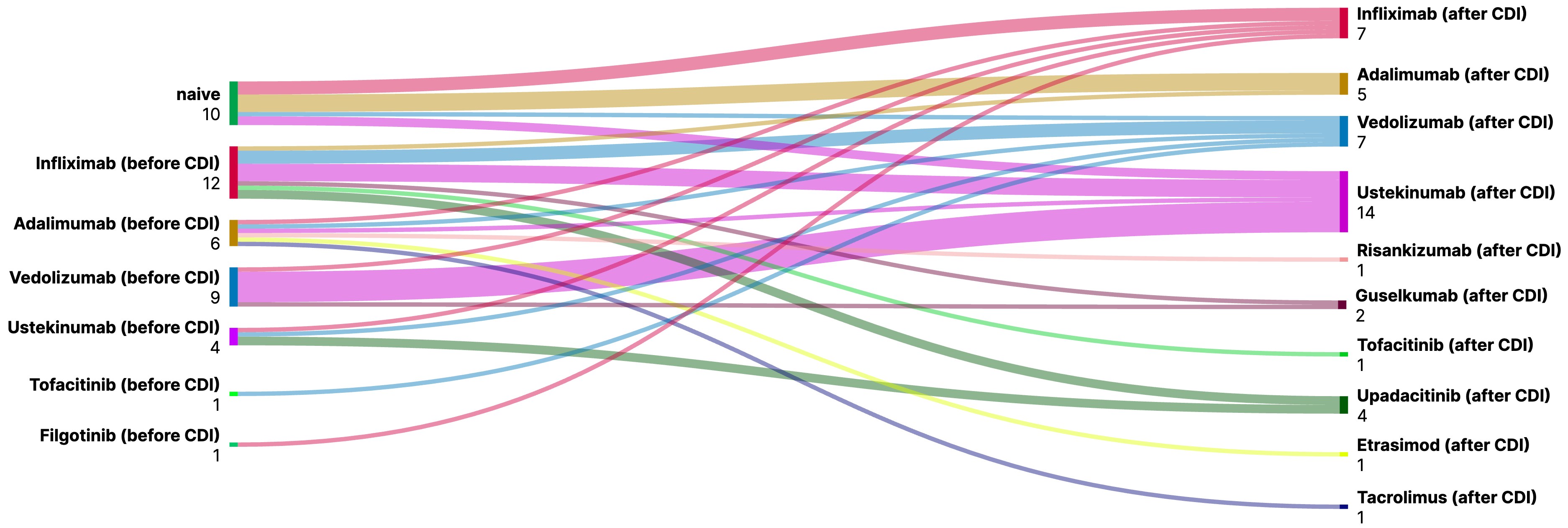

Supplement: jjaf056_suppl_Supplementary_Figure_S2 [file jjaf056_suppl_supplementary_figure_s2.jpeg]
