# Supplementary material for: Fidaxomicin for Clostridioides difficile infection in patients with inflammatory bowel disease: a multicenter retrospective cohort study
Source: J Crohns Colitis. 2025 Apr 1;19(5):jjaf056. doi: 10.1093/ecco-jcc/jjaf056 (PMC12060865; doi:10.1093/ecco-jcc/jjaf056)
Supplement: jjaf056_suppl_Supplementary_Table_S1 [file jjaf056_suppl_supplementary_table_s1.docx]

| Diagnostic test(s) | n |
| --- | --- |
| - GDH antigen test | 7 |
| - NAAT for toxigenic *C. difficile* | 5 |
| - Toxin enzyme immunoassay | 5 |
| - GDH antigen test + NAAT for toxigenic *C. difficile* | 26 |
| - GDH antigen test *+* toxin enzyme immunoassay | 31 |
| - NAAT for toxigenic *C. difficile* + toxin enzyme immunoassay | 22 |

**Supplementary table 1.** Microbial diagnostic testing for CDI.

GDH, glutamate dehydrogenase; NAAT, nucleic acid amplification test
